# Hope, Coping and Eco-Anxiety: Young People’s Mental Health in a Climate-Impacted Australia

**DOI:** 10.3390/ijerph19095528

**Published:** 2022-05-02

**Authors:** Hasini Gunasiri, Yifan Wang, Ella-Mae Watkins, Teresa Capetola, Claire Henderson-Wilson, Rebecca Patrick

**Affiliations:** School of Health and Social Development, Faculty of Health, Deakin University, Burwood, VIC 3125, Australia; yifan.wang@deakin.edu.au (Y.W.); ella-mae.watkins@outlook.com (E.-M.W.); teresa.capetola@deakin.edu.au (T.C.); claire.henderson-wilson@deakin.edu.au (C.H.-W.); rebecca.patrick@deakin.edu.au (R.P.)

**Keywords:** climate change, young people, mental health, social media

## Abstract

(1) Background: In Australia, young people are one of the most vulnerable populations to the mental health impacts of climate change. The aim of this article was to explore mental health promotion issues related to climate change for young people in Australia. (2) Methods: An exploratory mixed-method approach, co-led by young people, was used to engage young people living in Australia aged 18–24 years in semi-structured interviews (N = 14) and an online survey (N = 46). Data were analysed thematically and with descriptive statistics. (3) Results: Findings indicated that negative impacts included worry, eco-anxiety, stress, hopelessness/powerlessness and feelings of not having a voice. Several mediating factors, in particular social media engagement, highlighted the duality of mental health impacts for young people’s mental health. Positive impacts of climate action included feeling optimistic and in control. (4) Conclusions: This exploratory study contributes to an emerging field of public health research on young people’s mental health in a climate-impacted Australia. Climate change is a significant concern for young people, and it can negatively affect their mental health. The findings can inform the design of public health interventions that raise awareness of climate change-related mental health issues among young people and promote their participation in nature-based interventions, climate action and empowering social media engagement.

## 1. Introduction

“Australia is one of the most vulnerable developed countries in the world in the climate crisis” [1]. Climate change is already having catastrophic impacts on public health [2]. Research Australia [3] established that nearly two-thirds of Australians nominate increased mental health issues related to climate change as the greatest risk to health.

Climate change can directly and indirectly impact mental health [2,4]. Direct impacts of climate change, such as extreme weather events, can impact the prevalence and severity of mental health issues including traumatic stress, depression and anxiety disorders [4]. Climate change affects mental health indirectly via impacts on physical health and disruptions to the social and built environment. For example, loss and disruption of housing arising from extreme weather events may result in increased rates of depression in affected communities [4]. Two recent scoping reviews confirm the urgency of the climate crisis for mental health globally and the need for research addressing all five WHO global research priorities for protecting human mental health, especially for priority population groups such as youth [5,6].

The psychological effects of climate change are intensified for priority populations including young people. Children and young people exposed to extreme weather events and the resulting family stress and disruptions to social support networks are at higher risk of developing mental health problems, including post-traumatic stress disorder, depression, anxiety, sleep disorders and substance abuse [4]. A recent survey found that nearly half of young Victorians reported intense frustration, anxiety, sadness and anger related to climate change, which impacts mental health issues [7]. According to Sustainability Victoria [8], 73% of young people aged 15–24 years recognized health as one of the main ways they can be affected by climate change.

Climate action refers to individual, or organisational level, deliberate efforts and engagement to reduce greenhouse gas emissions and increase resilience to climate change impacts [9]. Whilst young people are among the most vulnerable to the impacts of climate change, they are powerful supporters for change [10]. Australian youth-led organisations such as the Australian Youth Climate Coalition and social media channels (e.g., School Strike 4 Climate) are active agents for climate action towards a more healthy, just and sustainable future [11]. International evidence suggests that young people’s engagement in climate action may have mental health benefits arising from social inclusion and active coping [12,13]. Hence, there appears to be a duality within the climate change experiences of young people and further research is required to illuminate young people’s experiences in a climate-impacted Australia.

Climate action can be conceptualised as a form of coping. Ojala and colleagues’ [13] study (N = 705) with Swedish children and adolescents conceptualised three strategies for coping. Problem-focused coping was seen as helpful in reducing the cause of the problem, while meaning-focused coping described beliefs, values and goals associated with positive feelings in young people dealing with the concern and worry associated with threats of climate change [13]. Finally, de-emphasizing the seriousness of climate change included avoidance, denial, distancing and a belief that climate change is exaggerated. It is related to lower environmental efficacy and fewer long-term mental health benefits [13].

This paper presents new insights on the link between climate change and mental health among young people. The duality of the climate change experience is explored; positive impacts of engaging in climate change action and negative impacts of climate change are identified, and coping strategies that young people are using are highlighted. Key issues associated with young people’s engagement with climate change on social media are explored. Understanding the link between climate change and young people’s mental health is paramount to developing public health interventions in an increasingly climate-impacted Australia.

## 2. Materials and Methods

An exploratory mixed-method approach was used to identify climate-related mental health promotion issues among young people aged 18–24 years in Australia. The study was undertaken with human research ethics approval from [BLINDED FOR REVIEW].

Convenience and criterion sampling strategies were used to recruit participants (N = 46) to complete a quantitative cross-sectional online survey hosted on Qualtrics from 13 July to 3 August 2020. The recruitment of participants involved two steps. Firstly, young people engaged in climate action were reached through the researchers’ existing networks via email and social media. Secondly, managers of youth and health organisations were invited to advertise for study participants in the general young people population through their networks (e.g., social media post and/or emailed advertisement in a newsletter). A mix of young people engaged in climate action and those from the general population was deemed important to minimise bias, generate a cross-section of young people’s perspectives and highlight any major differences. As the researchers did not have access to organisation or network databases, precise reach and participation rates of the online survey could not be obtained [14]. The final sample included 21 young people involved in climate change/environmental networks or activities and 25 young people from the general population. The mean age was 21 (SD = 1.92). The final sample was representative in terms of young people involved in climate change networks/activities and the general population not involved in climate change networks/activities.

Purposeful criterion sampling strategies were used to recruit participants (N = 14) engaged in climate action in key-informant interviews. Criterion sampling of young people engaged in climate actions provided an important qualitative component to the themes generated in the survey. Recruitment was conducted via social media and electronic news items through the researchers’ network of environmental and health organisations and [BLINDED FOR REVIEW] University. Fourteen young people who were involved in climate change activities and networks responded to the invitations and were recruited for the interviews.

The online survey presented 13 questions including opinions about mental health impacts of climate change, feelings associated with participation in different climate/environmental activities and feelings related to talking about climate change in different areas of life. The survey consisted of validated and new scales and was pre-tested by young people and academics.

The initial round of semi-structured interviews (N = 6) explored the survey themes in more depth. The interview protocol included questions on negative mental health impacts of climate change, positive mental health impacts of engaging in climate action as well as coping strategies. The second round of interviews (N = 8) included additional questions related to engagement with climate change content on social media. Interviews were led by two researchers aged under 25 years and ranged between 10 and 40 min. Interviews were conducted by N = 14 Zoom and were digitally recorded.

Quantitative data analysis was undertaken with Excel and STATA 16.0 with descriptive analysis. Qualitative data analysis was undertaken sequentially using thematic analysis techniques combined with inductive and deductive approaches [15]. The lead qualitative researcher read through the interview data and coded the first six transcripts. Codes from each interview were then compared in a table to find common themes and data with similar codes were collated into subthemes. The theme of “duality of experience” was identified and the major theme of “social media” was pursued in the next round of interviews. The data from the additional interviews were subject to thematic analysis and incorporated into theme tables. Another qualitative researcher coded these eight transcripts. The final step involved deductive analysis which involved the application of themes to the research questions. Both data sets were triangulated and interpreted in relation to existing literature from the fields of psychology and health promotion. The themes/results from the survey validated the findings from the interviews about mental health impacts of climate change on young people involved in climate change activities and the impact of social media on their mental health.

## 3. Results

### 3.1. Taking Its Toll: Negative Impacts of Climate Change on Young People’s Mental Health

**Figure 1:**
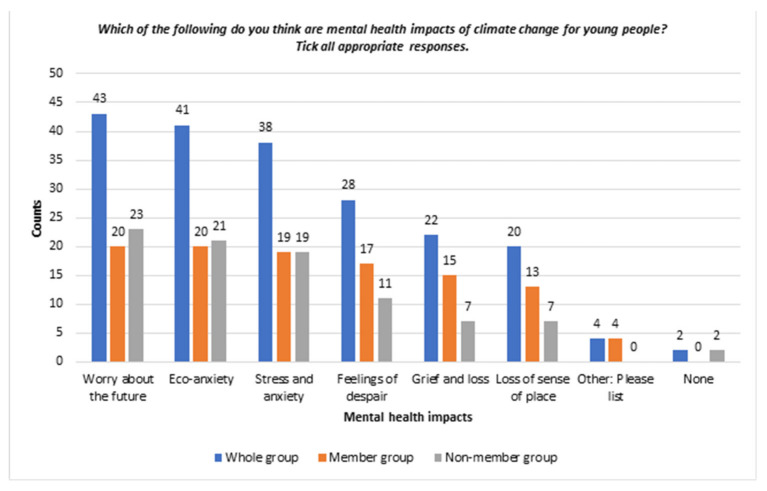
Bar chart of young people’s perceptions on negative mental health impacts of climate change—whole sample and between groups.

This section demonstrates how issues related to climate change negatively affected young people’s mental health. Figure 1 presents the participants’ opinions about the mental health impacts of climate change on young people.

*Worry about the future*. The survey demonstrated that 43 (93%) participants thought “worry about future” could be a mental health impact of climate change for young people. The mean of “worried” about climate change was 3.22 (“almost never = 1”, “sometimes = 2”, “often = 3”, “almost always = 4”), the highest frequency among all the negative “feelings” measured. The mean of “worried” in the member group was higher than in the non-member group: 3.76 vs. 2.76. Worry was identified as a common negative mental health impact of climate change by several interviewees.

*…And you go, “oh my gosh, in ten years I’ll be 30 some of these kids will only be in their 20 s”. They’ll be like what’s gonna happen then. So, I think worry is a huge one. [Interview participant 5]*

*Eco-anxiety.* A total of 41 out of 46 (89%) participants chose “eco-anxiety” as a likely mental health impact of climate change. A higher percentage of participants in the member group selected “eco-anxiety” (*n* = 20, 95% vs. *n* = 21, 84%) than in the non-member group.

Eco-anxiety was identified by some of the interviewees as a significant negative mental health impact which starts to emerge in young people when they start thinking about the future.

*It’s a massive concern because it is such a massive issue that seems like in a lot of places just being shoved to the ground and no-one cares. [Interview participant 6]*

*Stress and anxiety.* “Stress and anxiety” was perceived to be a mental health impact of climate change by 38 out of 46 (83%) survey participants. A higher percentage of participants selected “stress and anxiety” as a mental health impact in the member group than in the non-member group (*n* = 19, 90% vs. *n* = 19, 76%).

Interviewees also spoke of how stress and anxiety could result in distancing oneself from an issue.

*I think for me though stress and anxiety around a particular subject causes me to actually distance myself from that subject... [Interview participant 4]*

*Hopelessness and powerlessness.* Furthermore, the interview participants felt hopeless and powerless because they were not in positions to mitigate climate change impacts.

*But we’re not in positions that we can really do that much to impact it beyond making personal choices to be more sustainable, use re-usable items and all that kind of stuff. [Interview participant 6]*

Several of them mentioned they felt their voice were not heard, even though young people are aware of climate change and its impacts.

*...they’re aware of climate change, they’re aware of the effects it’s going to have, and I think they’re worried because they don’t feel like their voice is heard. [Interview participant 3]*

### 3.2. Taking Climate Action: Positive Impacts on Young People’s Mental Health

**Figure 2:**
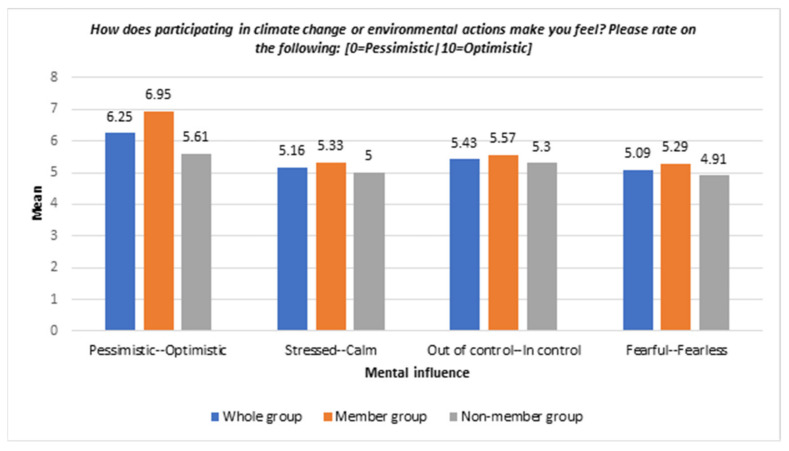
Bar chart of positive mental health impacts via different environmental activities—whole sample and between groups.

The participants’ feelings when engaged in climate action are presented in this section. Figure 2 demonstrates how climate or environmental actions influenced participants’ mental health. 

*Optimistic.* The survey demonstrated that young people felt more optimistic (mean: 6.25) when they participated in climate change or environmental actions. Notably, the mean of optimistic feelings in the member group was higher than in the non-member group (mean: 6.95 vs. 5.61).

Several of the interviewees communicated that engaging in climate change action and seeing their actions having a positive impact on others gave them a sense of hope over the climate crisis and made them feel good:

*I guess it takes that positive aspects of that sense of hope but like a really good level. (Laughs) Like “whoo!” the best level. [Interview participant 5]*

*Calm.* The survey found that participants felt marginally calmer (mean: 5.16) when they participated in climate change or environmental actions. The member group felt calmer than the non-member group, who were neutral (mean: 5.33 vs. 5).

*In control.* The survey showed that participants felt more in control (mean: 5.43) when they participated in climate change or environmental actions. The data tends to the positive side regardless of whether the individual was in the member group or non-member group.

*Fear.* Lastly, the survey suggested that participants felt more fearless (mean: 5.29) when they participated in climate or environmental actions compared to the non-member group.

The interview participants also added that taking action on climate change helped them feel like (a) they are part of the solution, (b) they are being heard and that (c) they are capable of making a difference:

*...then you can be a part of the solution um, I think it’s a big thing knowing that what you do can make a difference. [Interview participant 5]*

### 3.3. Talking Climate Change: Experiences in Different Parts of Young People’s Lives

This section presents the impacts of climate change on different areas of life. The impact of social media on the research participants’ mental health related to climate change is highlighted as a duality of the climate change experience.

**Figure 3:**
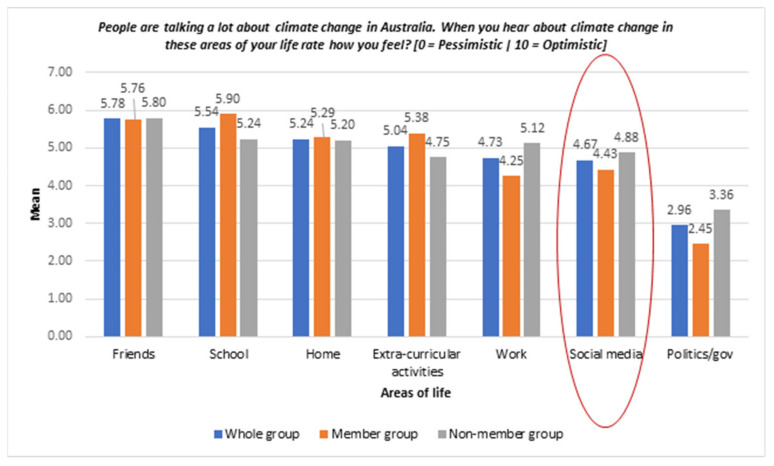
Bar chart of feelings related to talking about climate changes in different areas of life—whole sample and between groups. Red circle highlights how young people feel when they hear about climate change on social media.

Figure 3 presents participants’ responses to talking about climate changes in different areas of life. Mean scores greater than 5 indicate optimistic feelings. These included “with friends my age”, “at university or school”, “at home” and “in my extra-curricular activities”. Conversely, the mean scores of the other three areas of life including “at work”, “on social media” and “in politics and government” were lower than 5.

Interviewees spoke about how negative social media stories about climate change act as a detractor for young people’s mental health, including feelings of hopelessness:

*…the reality is there’s always gonna be negative new stories or things that are [on social media], I guess, back steps to what you are trying to achieve. And I think if you are constantly updated with that it makes you feel, um, that’s when you start to go like, “Oh great. Like what am I even trying to do? Is it, what I’m doing, is it having an effect?”. [Interview participant 6]*

Several interviewees noted they have experienced anxiety when viewing the impacts of climate change on social media. Learning of global climate events such as record high temperatures, rising sea levels and the melting of ice caps was identified as anxiety inducing. One participant shared the following:

*I do get a lot of climate anxiety when I read the stuff about how June was the hottest month, I’m like yeah I can’t do anything about that. [Interview participant 7]*

Social media can provoke feelings of guilt. The interviewees reported that some organisations on social media are influencing them to feel guilty when they do not have the capacity to partake in climate activism. They felt ashamed for not being able to donate money to causes or participate in sustainable behaviours:

*…they might just say ‘go vegan or vegetarian, it’s bad if you eat meat’ and that can make people feel quite guilty but without really completely understanding why and how it can be related to climate change. [Interview participant 11]*

Accessing climate information on social media at times can be overwhelming for young people. Content depicting animal suffering and negative narratives of the climate crisis was identified as being too confronting to continue to observe:

*…it’s a bit intense so I’m trying not to follow it, as much as it’s so important I don’t want to like dull my feed out by the negative impacts of climate change. [Interview participant 8]*

Paradoxically, social media also played a role in promoting positive mental health. Positive news stories showing people who care about the environment and people taking action helped to make young people feel good:

*…you see the positive stories about changes being made, about countries shifting to more reusable, sustainable sources and seeing that people are caring and are making a change. [Interview participant 6]*

Moreover, interviewees identified that social media can also provide a platform for young people’s voices and facilitate awareness raising of climate change.

*We were raised with all this [social media] and it allows you to get your voice out there. [Interview participant 9]*

*Yeah, I’d love to bring more awareness and you know show people what little things they can do, or what their current actions might impact on in the future if they are positive or negative. [Interview participant 1]*

Several interview participants mentioned that social media is powerful because (a) it is helpful in finding evidence on climate change action and (b) it gives people control and immediate contact or exposure, worldwide:

*You can be speaking to people on the other side of the globe that you’d never interact with at any other point in your life and you’re able to build a community and a network... [Interview participant 6]*

### 3.4. Coping Strategies

**Figure 4:**
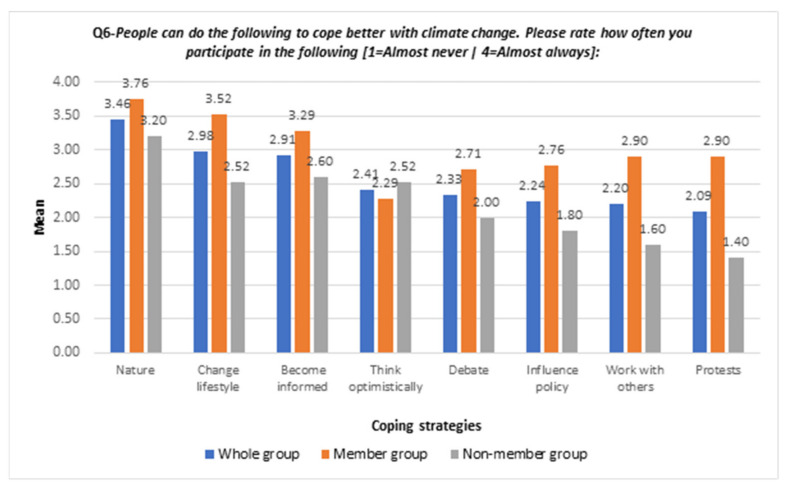
Bar chart of use of coping strategies between groups—whole sample and between groups.

Figure 4 refers to the use of coping strategies by the survey participants. For the whole group, the highest participation or use of coping strategy was “contact with nature” (mean: 3.46) and the lowest frequency was “participate in climate and environmental protests” (mean: 2.09).

Comparing the member group and non-member group, the mean of “thinking optimistically” as a coping strategy in the member group was less than in the non-member group (mean: 2.29 vs. 2.52). The means of coping strategies in the member group, which were more than in the non-member group, included “contact with nature” (mean: 3.76 vs. 3.20), “change lifestyle” (mean: 3.52 vs. 2.52), “become informed” (mean: 3.29 vs. 2.60), “debate” (mean: 2.71 vs. 2.00), “influence policy” (mean: 2.76 vs. 1.80) and “work with others” (mean: 2.90 vs. 1.60). Notably, the mean of “participate in climate and environmental protests” in the member group was twice the mean of the non-member group (mean: 2.90 vs. 1.40).

Several participants reported the importance of contact with nature for coping and as a motivator for climate action. These quotes highlight this:

*I know I feel much better when I’m riding out in the wild or when I’m surfing, whatever. So, not being able to do those things I know is going to impact me, heavily, down the track, progressively, progressively, progressively. [Interview participant 3]*

*Well, I live in [City A], which is very close to the coast and it was important, for me, to ensure that remained protected, I guess. [Interview participant 2]*

Social media was probed as a major theme in interviews. Participants indicated they use social media to become informed, learn about sustainable behaviours, participate in debate and influence policy and therefore practice activism. This participant stated that they do this in the realm of social media to become more conscious and critical in their thinking and to create meaningful and deliberate positive changes:

*“Probably to see where I could fit in make a change like you use social media as a vector by which to do things in real life, I guess.” [Interview participant 7]*

Several participants provided suggestions for combating the differing social media feeds consisting of both positive and negative information. These young people identified numerous instances in which they view polarising climate information and highlighted the importance of creating a balanced feed:

*“I guess like having the courage to just get rid of that sort of negativity and finding that balance between the negative stuff and realistic issues that you, want to be aware of, but not like going too deep down the rabbit hole where you can just sort of get into this doom and gloom sort of mindset and feel like nothing is good.” [Interview participant 12]*

## 4. Discussion

Burke, Sanson and Van Hoorn [12] reported that young people express fear, anxiety and worry about the impact of climate change on their future. Similar concerns were reported by this study’s participants and ranged from worry through to eco-anxiety, suggesting a continuum of intensity of feelings. The participants in this study worried about the future and the limited time remaining to effectively mitigate the impacts of climate change.

Participants also reported feelings of hopelessness and powerlessness because they were not in positions to mitigate climate change impacts. This is significant because, as Pihkala [16] warns, meaninglessness and powerlessness in life can negatively impact young people’s mental health. Concerns about the magnitude of climate change impacts and a bleak future generated feelings of stress and anxiety among the study participants. As in the current study, Clayton [17] asserts that stress and anxiety are a major threat to mental health wellbeing and can emerge due to the uncertainty of climate change.

Engaging in climate action can help young people manage their anxieties about the future and transform their feelings into optimism and determination [18]. The current study suggests that young people feel optimistic, calmer and in control when they take climate action. Moreover, recognising that climate action has a positive impact on others instilled a sense of hope and optimism in the study participants. Sanson, Van Hoorn and Burke [18] found that climate action and hope mutually reinforce each other; hope can inspire climate action, but climate action can also inspire hope.

Having the ability to make changes or taking action, that is, a “sense of agency”, as well as personal resilience, also promotes mental health and wellbeing. Some of the participants believed in their ability to make changes or take climate action, and in turn, this helped them feel optimistic about the future. Young people’s sense of agency appeared to support climate action, which helped rebuild the hopes for their future when faced with difficult situations. These findings are in keeping with Sanson, Van Hoorn and Burke’s [18] view who argue that resilience and agency of young people can be developed by encouraging and supporting them in climate action. When young people take a meaningful role in climate change action, it offers psychological protection by helping them to feel more hopeful, more in control and more resilient [18].

Social media is ubiquitous in young people’s lives [19]. It can both alleviate negative mental health as well as act as a contributor to it. We found that when young people interact with negative news stories about climate change on social media, they are more exposed to negative mental health effects. Hodson, Dale and Peterson [20] found that negative reporting of climate change can lead to hopelessness and decreased feelings of agency. Conversely, the current study found that constructively framed news stories on climate action had a positive effect on young people’s social and mental health wellbeing. Moreover, they viewed social media as an accessible and powerful platform for their voice and awareness-raising actions on climate change. These findings concur with the study of Pearce et al. [21], that social media enables engagement with diverse audiences, knowledge mobilization and transfer while bridging disparate communities.

Problem- and meaning-focused coping mechanisms are positively associated with young people’s ability to cope with climate change [13]. Concern about the negative impacts of climate change on the planet and people was the main reason for involvement in climate change action in the current study. According to Ojala and Bengtsson [13], meaning-focused coping reinforces beliefs, values and goals. This leads to more positive feelings in young people which counteract the overly worried and heightened concerns associated with climate change, without denying the reality. This coping strategy is positively associated with environmental engagement and efficacy, higher life satisfaction and wellbeing and optimism [13]. Problem-focused coping can also alleviate negative mental health impacts; however, unless this is accompanied by congruent environmental values, young people do not feel buoyant and positive. They can become overwhelmed by personal responsibility and the enormity of the problem and this can be harmful to their mental health.

The study explored different settings or activities in young people’s lives where they felt more optimistic about climate change issues and where they enacted “coping”. Nature contact had the most positive impact, which resonates with extensive literature concerning the mental health benefits of nature, including feelings of pleasure and tranquillity, and reducing negative feelings, such as anxiety and anger [22,23].

Nature and social media were highlighted as two key settings for promoting climate-related mental wellbeing among young people. Nature as a setting for young people’s mental health promotion is an area of practice which could be expanded through public health investment in initiatives such a nature/green scripts and other nature-based interventions such as bush therapy [24,25]. The study also identified a range of individual and community level strategies that are potentially helpful for young people’s mental and social health. The implication for public health is that a multi-faceted approach to intervention design, including a combination of individual, group, community and organisational level strategies, is required to be effective in promoting mental health in the context of climate change for young people. Young people-led and co-designed social media campaigns have the potential to engage young people in solutions, promote a sense of agency and active coping and in turn promote mental health. WHO considers climate change a social determinant for mental health [26]. In line with WHO’s five global research priorities for protecting human health from climate change, this research contributes to two of the under-researched areas: identifying the most effective interventions to protect health from climate change and guiding health promotion mitigation and adaptation decisions in other sectors [6]. The practical value of this current research is in identifying the mental health-promoting aspects of young people’s involvement in climate action and contact with nature, which has dual mitigation and adaptation benefits. Moreover, articulating the dual response to young people’s engagement with social media as both a protective and causal determinant of mental health facilitates much needed dialogue with sectors external to health, in advocating for responsible and proactive social media platforms to build young people’s mental health wellbeing.

This study is one of the few exploratory mixed-method Australian studies co-led by young people which gives voice to young people’s concerns about mental health in relation to climate change. The quantitative survey had a good completion rate and an even split between members of climate change organizations and non-members. A limitation is the small sample size. A more representative sample may be used in the future to verify initial insights from this study. The COVID-19 pandemic necessitated several changes to the research methodology, e.g., online interviews rather than face to face.

## 5. Conclusions

While climate change poses serious threats to population health, young people are particularly vulnerable to climate-related mental health issues. Utilising both quantitative and qualitative research methods co-designed by young people, the current study found that young people’s mental health and wellbeing are negatively impacted by climate change as expressed by anxiety, eco-anxiety, hopelessness, stress and existential loss. Young people also related feeling a lack of control and powerlessness in response to government inertia, community inaction and dystopic climate representations through social media. However, feelings of hope and mobilisation were also apparent through young people’s exposure to optimistic climate reports on social media, connecting with nature and through proactive climate action. A duality of responses to social media mediated by climate reporting provides leverage points for climate activists and public health practitioners for enhancing mental health and wellbeing in young people by emphasising positive climate action. Connecting with nature provides young people with an affinity to the natural world, a direct link between climate action and wellbeing as well as a reprieve from climate-induced stress and climate action fatigue. Going forward, public health practitioners and climate activists can engage young people in positive climate-related mental health promotion through nature-based interventions and climate action. Undertaking a pledge for planetary health can also unite health professionals in a climate-impacted world [24] and unify approaches to promote positive mental health wellbeing for young people.

## Data Availability

Data is contained within the article.
